# Evaluation of hepatic antioxidant capacities of *Spirogyra neglecta* (Hassall) Kützing in rats

**DOI:** 10.2478/intox-2013-0024

**Published:** 2013-09

**Authors:** Tarika Thumvijit, Waristha Thuschana, Doungporn Amornlerdpison, Yuwadee Peerapornpisal, Rawiwan Wongpoomchai

**Affiliations:** 1 Department of Biochemistry, Faculty of Medicine, Chiang Mai University, Chiang Mai, Thailand; 2 Department of Biology, Faculty of Science, Chiang Mai University, Chiang Mai, Thailand; 3 Faculty of Fisheries Technology and Aquatic Resources, Maejo University, Chiang Mai, Thailand

**Keywords:** antioxidant, green algae, *Spirogyra neglecta* (Hassall) Kützing

## Abstract

Free radicals are one of the causes of chronic and degenerative diseases. Antioxidants can protect the progression of free radical mediated disorders. The aim of this study was to evaluate the antioxidant activity of *Spirogyra neglecta* (Hassall) Kützing in rats. The rats were divided into 5 groups. Group 1 served as control. Groups 2 and 3 were administered hot water extract of *S. neglecta* at 50 and 200 mg/kg bw, respectively, while groups 4 and 5 were fed 1% and 4% *S. neglecta* mixed diet, resp., for 13 weeks. Antioxidant enzymes were evaluated in livers of the rats. The activities of catalase and glutathione reductase were significantly increased in the group fed 50 mg/kg of the extract, compared with the control group. Glutathione peroxidase activity was also significantly higher in the group fed 50 and 200 mg/kg of the extract. The study suggests that *S. neglecta* may enhance antioxidant systems in the rat liver.

## Introduction

Reactive oxygen species include the hydroxyl radical, the superoxide anion radical, hydrogen peroxide and various lipid peroxides (Lee & Min, 2004). They are generated in all living organisms. These free radicals can react with various biomolecules in cells, leading to cell damage and death. This phenomenon appears to be a major contributor in chronic and degenerative diseases such as cancer, diabetes, arthritis, and atherosclerosis (Sapakal *et al.,* 2008). Antioxidants can either trap or destroy free radicals and other reactive oxygen species, thus preventing oxidative stress-related diseases (Ak & Gülçin, 2008). The antioxidant system includes endogenous enzymes such as superoxide dismutase, glutathione peroxidase, glutathione reductase and catalase, as well as non-enzymatic anti-oxidants such as glutathione, ascorbic acid and tocopherol (Sapakal *et al.,* 2008). Consumption of fruit and vegetables rich in natural antioxidants is associated with the prevention of degenerative diseases (Ferrari, 2004; Veerapur *et al.,* 2009).

Algae are a great source of natural compounds that are widely known and consumed in Asian countries. Numerous studies have investigated compounds found in algae for their antibiotic, antiviral, antioxidant, anti-inflammatory and cytotoxic activities (Lordan *et al.,* 2011). *Spirulina* is a blue-green microalga from the Cyanobacterium gender and contains a phycocyanin with pharmaceutical and antioxidant properties (Estrada *et al.,* 2001). Brown algal polyphenols function as antioxidants, antibacterial and anti-fungal compounds (Kuda *et al.,* 2007; Shibata *et al.,* 2006). Seaweeds are known to contain reactive antioxidant molecules, such as ascorbate, glutathione, carotenoids, catechins and polysaccharides (Yuan *et al.,* 2005). Polysaccharides in various algae have been demonstrated to act as free radical scavengers *in vivo* and *in vitro* (Jiao *et al.,* 2011). However, most previous studies have focused on seaweed, blue-green, red and brown algae. There is little information concerning freshwater green algae and their pharmacological and medical application.

*Spirogyra* spp. are filamentous freshwater green algae. These algae are consumed as food in northern Thailand. *Spirogyra neglecta* (Hassall) Kützing contains high amounts of protein, carbohydrate, fat, sulfate and dietary fiber (Phinyo *et al.,* 2012). *S. neglecta* is beneficial to the environment via removing Pb^2+^ from polluted water (Hussain *et al.,* 2009). It has also pharmacological properties. *S.neglecta* extract can inhibit gastric ulcer formation induced by physical and chemical stress in rats. It also showed hypolipidemic and hypoglycemic abilities in type 2 diabetic rats induced by streptozotocin and high fat diet (Lailerd *et al.,* 2009). The mechanism responsible may be related to *S.neglecta*'s antioxidant properties. The present study thus aimed to determine the *in vivo* antioxidant effects of *Spirogyra neglecta*.

## Materials and methods

### Chemicals

β-dihydronicotinamide adenine phosphasedinucleotide (β-NADPH) was purchased from Oriental Yeast Co., Ltd. (Tokyo, Japan). Cupric chloride (CuCl_2_) was purchased from BDH (BDH Chemicals Ltd, Poole, England). All other chemicals used were purchased from Sigma-Aldrich Co. (St. Louis, Mo, USA).

### Materials

*Spirogyra* spp. was collected from the cultivation pool of Na Kuha village, Saun Kuen Sub district, Muang District, Phrae Province, Thailand, during October and November 2010. The fresh algae were identified and authenticated according to the morphology both of vegetative cells and sex cells and their habitat by the method of John *et al.* (2002). The algae were extracted with distilled water at 100 °C for 2 h. The hot water extract was filtered with Whatmann filter paper no. 1 and lyophilized with a freeze dryer.

### Chemical composition analysis

The alga *S. neglecta* was analyzed for total carbohydrate, sulfate, chlorophyll and total phenolic compounds. Carbohydrate was determined by the phenol-H_2_SO_4_ method using glucose as the standard (Dubois *et al.,* 1956). Sulfate was measured by the method of Craigie and Wen (1984) using K_2_SO_4_ as standard. Total phenolic compounds were determined by the Folin-Ciocalteu method using gallic acid as standard (Emmons *et al.,* 1999). Chlorophyll was determined according to the method described by Proctor (1981).

### Animals

Male Wistar rats weighing between 120–150 g were purchased from the National Laboratory Animal Center, Salaya, Nakorn Patom, Thailand. They were housed in the animal care center of the Faculty of Medicine, Chiang Mai University, under controlled environmental conditions of 24 °C and 12 h light-dark cycle. The experimental protocol was approved by the Animal Ethics Committee of the Faculty of Medicine, Chiang Mai University.

### Experimental protocol

All rats were randomly divided into 5 groups of 6 animals each with treatment groups as follows: Group 1 served as control, receiving distilled water and basal diet, Groups 2 and 3 received 50 and 200 mg/kg body weight of *S. neglecta* extract via intragavage for 13 weeks, respectively, Groups 4 and 5 were fed with *S.neglecta* mixed diets at 1% and 4% for 13 weeks, respectively. All animals were sacrificed at the end of the 13^th^ week by diethyl ether. Liver samples were removed for biochemical analysis. Blood samples were collected to determine liver function enzymes.

### Liver function tests

Alanine aminotransferase and aspartate aminotransferase enzymes in serum were measured spectrophotometrically using commercial Olympus kits (Olympus Corp., Tokyo, Japan).

### Determination of oxidative stress and antioxidant status in rat livers

#### Lipid peroxidation

The lipid peroxidation levels in livers were measured in terms of thiobarbituric acid reactive substance (TBARS) according the method of Fujiwara *et al.* (2003). The reaction mixture consisted of 10% liver homogenate, 50% trichloroacetic acid and 0.67% thiobarbituric acid aqueous solution. The reaction mixture was heated in a boiling water bath for 10 minutes. The tubes were placed on ice to stop the reaction, n-butanol was added, then mixed and centrifuged at 3,000 rpm for 20 minutes. The supernatant was measured for its absorbance at 532 nm. TBARS generation was calculated based on a standard curve of MDA.

#### Liver cytosol preparation

Frozen livers were homogenized with ice-cold buffer containing 1.5% KCl and 0.25 mM PMSF using a Polytron homogenizer. The homogenates were centrifuged at 10,000 g for 20 minutes at 4 °C. The supernatants were further centrifuged at 100,000 g for 60 minutes at 4 °C. The cytosols were stored at –80 °C until analysis. The protein content in rat liver cytosols was assayed via the Lowry method.

#### Glutathione

Glutathione was measured according to the method of Akerboom and Sies (1981) based on the reaction with 5,5’-dithiobis-2-nitrobenzoic acid (DTNB). The amount of glutathione was expressed as nmol/mg protein.

#### Superoxide dismutase (SOD)

This assay is based on the inhibition of nitroblue tetrazolium (NBT) reduction by superoxide radicals produced by the xanthine/xanthine oxidase system (Sun *et al.,* 1988). The SOD activity was expressed as U/mg protein.

#### Catalase

Catalase activity was determined by measuring the decomposition of hydrogen peroxide according to the method of Aebi (1984). Catalase activity was expressed as nmol of hydrogen peroxide reduced/min/mg protein.

#### Glutathione peroxidase (GPx)

GPx activity was determined by NADPH oxidation in a coupled reaction system containing *t*-butyl hydroperoxide (*t*-BHA) and oxidized glutathione (Nagalakshmi & Prasad, 2001). GPx activity was expressed as µmol of β-NADPH oxidized/min/mg protein.

#### Glutathione reductase (GR)

GR activity was determined according to the method of Carlberg and Mannervik (1983) by measuring the amount of NADPH consumed during the conversion of oxidized glutathione (GSSG) to reduced glutathione at 340 nm. GR activity was expressed as nmol of β-NADPH oxidized/min/mg protein.

### Statistical analysis

The experimental results are expressed as mean of 6 animals in each group±SD. The statistical significance of differences between groups was analyzed by one way analysis of variance (ANOVA) with LSD for *post hoc* tests.

## Results

**Figure 1:**
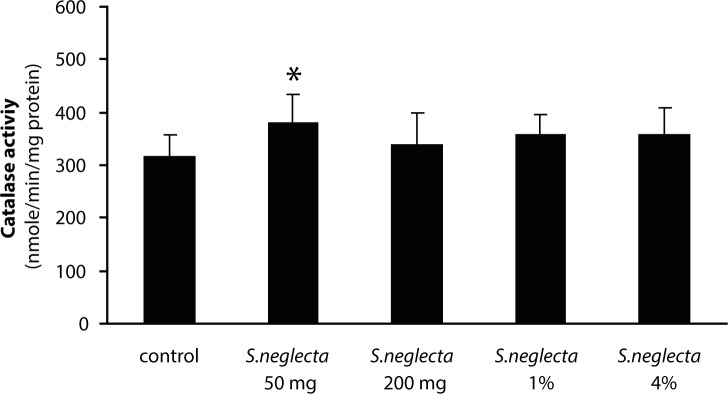
Effect of *S. neglecta* on hepatic catalase activity in rats. Results obtained from six rats of each group. Values are mean ± SD. *p<0.05, significantly different when compared with the control group.

**Figure 2:**
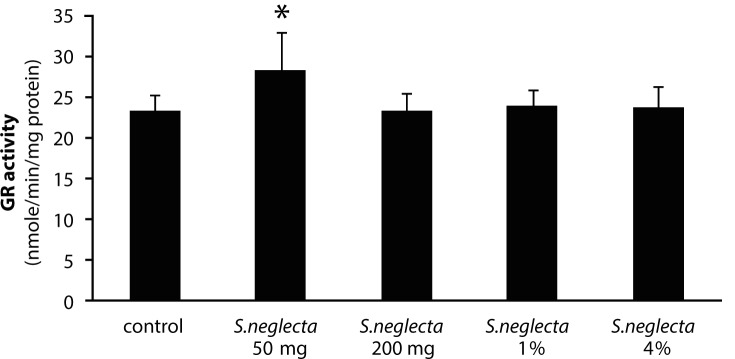
Effect of *S.neglecta* on hepatic glutathione reductase activity in rats. Results obtained from six rats of each group. Values are mean ± SD. *p<0.05, significantly different when compared with the control group.

**Figure 3:**
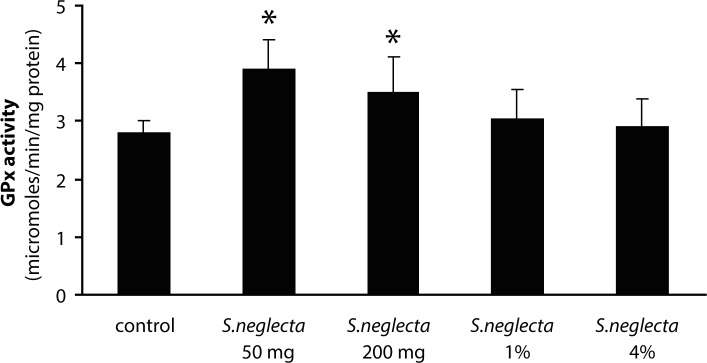
Effect of *S.neglecta* on hepatic glutathione peroxidase activity in rats. Results obtained from six rats of each group. Values are mean ± SD. *p<0.05, significantly different when compared with the control group.

**Figure 4:**
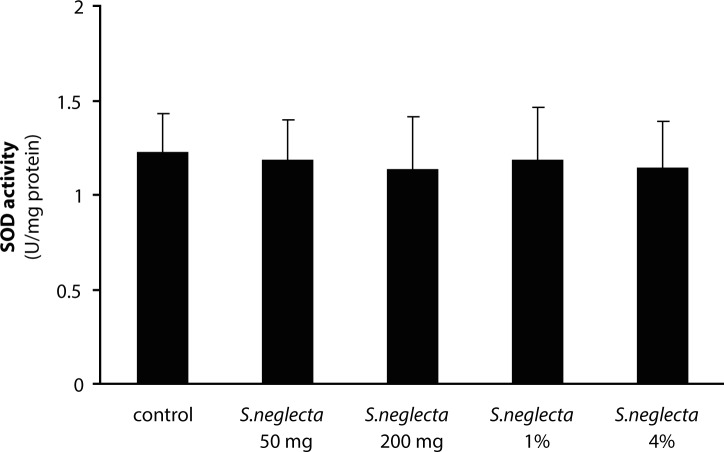
Effect of *S. neglecta* on hepatic superoxide dismutase activity in rats. Results obtained from six rats of each group. Values are mean ± SD.

The yield of hot water extract of *S. neglecta* was approximately 25%. Total carbohydrate and sulfate in *S. neglecta* were 33.94±1.46% and 1.08±0.01%, respectively. Chlorophyl content in dried *S. neglecta* and hot water extract from *S. neglecta* were 15.44 and 9.91 mg/g and total phenolic compounds were 72.98±14.34 and 151.02±15.39 mg GAE/g, respectively. Table 1 shows body weights and liver weights in *S.neglecta* treated and control groups. The food and water intake in all *S. neglecta* treated groups were not significantly different when compared with the control group. AST and ALT activities were not significantly changed in treatment groups as compared with the control group. No significant differences were observed in glutathione and TBARS levels in either treated group when compared with the control group (Table 2). Antioxidant enzymes were evaluated in livers of the rats. The activities of catalase and GR in the liver were significantly (*p<*0.05) increased only in the 50 mg/kg *S. neglecta* treated group (Figures 1 and 2). GPx activity was significantly (*p<*0.05) increased in livers of rats fed with 50 and 200 mg/kg of *S. neglecta* as compared with controls (Figure 3). SOD was not significantly changed in the treatment groups as compared with the control group (Figure 4).

**Table 1:** General appearance and liver weight of *S. neglecta* treated rats.

Groups	Food intake (g/rat/day)	Water intake (ml/rat/day)	Initial body weight (g)	Final body weight (g)	% body weight change absolutes	Liver weight (g)	Relative liver weight (%)
Control	21±3	32±12	101±4	428±10	324.4±18.4	13.3±2.7	3.0±0.4
*S. neglecta* 50 mg/kg	21±4	39±9	100±3	441±42	340.7±37.4	13.4±1.7	2.9±0.3
*S. neglecta* 200 mg/kg	23±4	37±11	100±4	461±43	360.8±37.6	13.5±1.5	2.9±0.5
1% *S. neglecta* mixed diet	23±3	33±8	102±4	467±49	358.4±38.2	12.7±1.3	2.9±0.3
4% *S. neglecta* mixed diet	22±3	31±8	103±4	435±17	324.9±21.7	13.1±1.6	3.1±0.4

Values are means ± SD, n=6

**Table 2:** Effects of *S. neglecta* on the level of serum AST and ALT activities and hepatic TBARS and glutathione in rats

Groups	AST (U/l)	ALT (U/l)	TBARS (nmole/mg protein)	GSH (nmole/mg protein)
Control	76±7	32±8	0.14±0.06	5.99±2.83
*S. neglecta* 50 mg/kg	99±22	30±4	0.15±0.04	7.29±1.57
*S. neglecta* 200 mg/kg	83±12	31±6	0.16±0.05	8.06±1.37
1% *S. neglecta* mixed diet	81±5	33±3	0.16±0.05	6.19±1.43
4% *S. neglecta* mixed diet	83±8	33±6	0.16±0.05	6.38±1.51

Values are means ± SD, n=6

AST: alanine aminotransferase, AST: aspartate aminotransferase, GSH: glutathione, TBARS; thiobarbituric acid reactive substances

## Discussion

Algae are alternative nutrient sources that contain abundant functional food ingredients. While many studies have shown the beneficial effects of marine algae, the present study is the first to provide information concerning the antioxidant activity of *S. neglecta*, a freshwater macroalga, in animal models. The administration of hot water extract of *S. neglecta* presented antioxidant properties in rats, while rats fed with *S. neglecta* mixed diet did not show induction of hepatic antioxidant enzymes.

Our previous studies showed *in vitro* antioxidant activity of warm (60 °C) water extract of *S. neglecta* (Peerapornpisal *et al.,* 2012). However, since the hot water extract of *S. neglecta* contains a higher phenolic content than does the warm water extract, the *in vivo* antioxidant activity of hot water extract of *S. neglecta* was determined in this study. The amounts of *S. neglecta* used in the analysis, which ranged from human daily consumption level to fourfold overdose, were not toxic to rats. Body and liver weights, daily consumption and liver function markers in serum were found to be comparable in treated and control rats.

Antioxidant enzymes play a crucial role in cellular defense oxidative stress. Catalase is an enzyme which converts hydrogen peroxide to water and molecular oxygen, thus preventing the formation of extremely dangerous hydroxyl radicals from hydrogen peroxide. It has long been known that hydroxyl radicals can destroy biomolecules leading to pathological alterations (Limon-Pacheco & Gonsebatt, 2009). Thus induction of catalase activity by *S. neglecta* treatment may result in a decrease in deleterious effects due to hydrogen peroxide. GPx plays an important role in protecting cells from free radicals generated by peroxide decomposition (Limon-Pacheco & Gonsebatt, 2009). GR is a ubiquitous NADPH-dependent enzyme protecting against oxidative damage within the cell by maintenance of appropriate levels of intracellular glutathione (Sapakal *et al.,* 2008). The enhancement of these antioxidant enzyme activities in livers of rats treated with hot water extract of *S. neglecta* indicated that *S. neglecta* may protect against cellular and tissue damage due to hydrogen peroxides, peroxides and hydroxyl radicals.

The antioxidant potentials of algae in human health have been discussed elsewhere (Cornish & Garbary, 2010). The major groups of antioxidant compounds in macroalgae are phenolic compounds, polyphenols, sulfated polysaccharides, carotenoids and vitamins. Phenolic compounds and polyphenols can chelate metal ions, prevent radical generation and indirectly modulate the activities and alter the expression levels of significant proteins, such as antioxidant and detoxifying enzymes (Ferguson, 2001; Ferguson *et al.,* 2004). Many studies have shown that the sulfated polysaccharides in algae possess various pharmacological activities including antioxidant activities (Jiao *et al.,* 2011). Zhang *et al.* (2003) reported that polysaccharide fractions from the alga *Porphyra haitanesis* (Rhodephyta) could increase antioxidant enzymes in aging mice. Song *et al.* (2010) demonstrated the inhibitory effect of polysaccharides extracted from the green alga *Bryopsis plumose* on superoxide radical and DPPH. In this study, we found that the total amount of phenolic compounds, total carbohydrates and sulfate of hot water extract were greater than those of raw materials. This may be one reason why the administration of the hot water extract of *S. neglecta* enhanced antioxidant systems in rat liver. Vitamin E and β-carotene, fat-soluble vitamins, and vitamin C, a heat unstable vitamin, are well-known antioxidants in algae. These antioxidant compounds might be excluded from this study due to the hot water preparation of *S. neglecta* extract. Furthermore, chlorophyl, green pigments found in algae and higher plants, exhibited various biological properties including anticancer and antioxidant activities (Ferruzzi & Blakeslee, 2007). The present study showed that the content of chlorophyl in dried *S. neglecta* was higher than in the hot water extract. This indicated that chlorophyl might not be the important antioxidant substances in *S. neglecta*. Further studies are needed to identify and determine polysaccharides and the other polar compounds of *S. neglecta* and their antioxidant activities.
